# DEPRESSION AND COGNITIVE DECLINE OF OLDER ADULTS IN THE TRANSITION TO WIDOWHOOD

**DOI:** 10.1093/geroni/igz038.3538

**Published:** 2019-11-08

**Authors:** Feng Zhao, Peter Martin, Gina Lee

**Affiliations:** Iowa State University, Ames, Iowa, United States

## Abstract

This study examined the trajectories of depression and cognitive function in the transition to widowhood and investigated the temporal and causal relationship between these two closely related constructs. Respondents were 1,822 widowed adults aged 51 to 91 from a restructured data set (Wave 3 to Wave 12) of the Health and Retirement Study. The results of cross-lagged panel analysis indicated a bidirectional relationship between depressive symptoms and cognition decline, but the effects of cognitive impairment at earlier time points on later depression were larger than the effects of previous depressive symptoms on later cognition. The latent growth curve analysis showed that the cognitive function declined over time, whereas the initial level of depressive symptoms first increased following widowhood and gradually decreased over time. Significant negative associations were found between the initial levels of depression and cognitive function (p < .001) and between the rates of change of these two variables (p = .025). Older adults tended to have lower initial level of cognitive function and they showed faster cognitive decline over time. Female respondents were more likely to report more depressive symptoms and higher cognitive function. White respondents were more likely to report fewer depressive symptoms and higher levels of cognitive function. Higher levels of education were protective for one’s cognitive function but not for depressive symptoms. The study highlighted the reciprocal relationship between depression and cognitive function following widowhood and pointed out that accelerated cognitive decline may precede elevated levels of depression.

